# Cauchy problem of the generalized Zakharov type system in $\mathbf{R}^{2}$

**DOI:** 10.1186/s13660-017-1306-2

**Published:** 2017-02-01

**Authors:** Shujun You, Xiaoqi Ning

**Affiliations:** Department of Mathematics, Huaihua University, Huaihua, 418008 China

**Keywords:** Zakharov system, Cauchy problem, existence, uniqueness

## Abstract

In this paper, we consider the initial value problem for a two-dimensional generalized Zakharov system with quantum effects. We prove the existence and uniqueness of global smooth solutions to the initial value problem in the Sobolev space through making *a priori* integral estimates and the Galerkin method.

## Introduction

In the recent years, special interest has been devoted to quantum corrections to the Zakharov equations for Langmuir waves in a plasma [1]. By use of a quantum fluid approach, the following modified Zakharov equations are obtained:

1 $$\begin{aligned} &iE_{t}+ E_{xx}-H^{2} E_{xxxx}=nE, \end{aligned}$$

2 $$\begin{aligned} &n_{tt}-n_{xx}+H^{2} n_{xxxx}= \vert E\vert ^{2}_{xx}, \end{aligned}$$

where *H* is the dimensionless quantum parameter given by the ratio of the ion plasmon and electron thermal energies. For $H=0$, this system was derived by Zakharov in [2] to model a Langmuir wave in plasma. The Zakharov system attracted many scientists’ wide interest and attention [3–14].

In this paper, we deal with the following generalized Zakharov system:

3 $$\begin{aligned} &iE_{t}+\Delta E-H^{2}\Delta^{2}E-nE=0, \end{aligned}$$

4 $$\begin{aligned} &n_{tt}-\Delta n+H^{2}\Delta^{2}n- \Delta \vert E\vert ^{2}=0, \end{aligned}$$

where $(E, n ): (x, t)\in\mathbf{R}^{2}\times\mathbf{R}$ and the initial data are taken to be

5 $$ E|_{t=0}=E_{0}(x),\qquad n|_{t=0}=n_{0}(x),\qquad n_{t}|_{t=0}=n_{1}(x). $$

To study a smooth solution of the generalized Zakharov system, we transform it into the following form:

6 $$\begin{aligned} &iE_{t}+\Delta E-H^{2}\Delta^{2}E-nE=0, \end{aligned}$$

7 $$\begin{aligned} &n_{t}-\Delta\varphi=0, \end{aligned}$$

8 $$\begin{aligned} &\varphi_{t}-n+H^{2}\Delta n-\vert E\vert ^{2}=0, \end{aligned}$$

with initial data

9 $$ E|_{t=0}=E_{0}(x),\qquad n|_{t=0}=n_{0}(x),\qquad \varphi|_{t=0}=\varphi_{0}(x). $$

Now we state the main results of the paper.

### Theorem 1.1

*Suppose that* $E_{0}(x)\in H^{l+4}(\mathbf{R}^{2})$, $n_{0}(x)\in H^{l+2}(\mathbf{R}^{2})$, $n_{1}(x)\in H^{l}(\mathbf{R}^{2})$, $l\geq0$. *Then there exists a unique global smooth solution of the initial value problem* (3)-(5).

$$\begin{aligned} &E(x,t)\in L^{\infty}\bigl(0,T;H^{l+4} \bigl( \mathbf{R}^{2} \bigr) \bigr),\qquad E_{t}(x,t)\in L^{\infty}\bigl(0,T;H^{l} \bigl(\mathbf{R}^{2} \bigr) \bigr) \\ &n(x,t)\in L^{\infty}\bigl(0,T;H^{l+2} \bigl( \mathbf{R}^{2} \bigr) \bigr), \qquad n_{t}(x,t)\in L^{\infty}\bigl(0,T;H^{l} \bigl(\mathbf{R}^{2} \bigr) \bigr) \\ &n_{tt}(x,t)\in L^{\infty}\bigl(0,T;H^{l-2} \bigl( \mathbf{R}^{2} \bigr) \bigr). \end{aligned}$$

The obtained results may be useful for better understanding the nonlinear coupling between the ion-acoustic waves and the Langmuir waves in a two-dimensional space.

## *A priori* estimates

### Lemma 2.1

*Suppose that* $E_{0}(x)\in L^{2}(\mathbf{R}^{2})$. *Then*, *for the solution of problem* (6)∼(9), *we have*

$$\Vert E\Vert ^{2}_{L^{2}(\mathbf{R}^{2})}= \bigl\Vert E_{0}(x) \bigr\Vert ^{2}_{L^{2}(\mathbf{R}^{2})}. $$

### Proof

Taking the inner product of (6) and *E*, then taking the imaginary part, we have

$$\begin{aligned} &\operatorname {Im}(iE_{t},E )=\operatorname {Re}(E_{t},E )=\frac{1}{2} \frac {d}{dt}\Vert E\Vert ^{2}_{L^{2}}, \\ &\operatorname {Im}\bigl(\Delta E-H^{2}\Delta^{2}E-nE,E \bigr)=0. \end{aligned}$$

Hence, we get

$$\frac{d}{dt}\Vert E\Vert ^{2}_{L^{2}}=0. $$

We thus get Lemma 2.1. □

### Lemma 2.2

Sobolev’s estimations

*Assume that* $u\in L^{q}(\mathbf{R}^{n})$, $D^{m}u\in L^{r}(\mathbf{R}^{n})$, $1\leq q,r\leq \infty, 0\leq j\leq m$, *we have the estimations*

$$\bigl\Vert D^{j}u \bigr\Vert _{L^{p}(\mathbf{R}^{n})}\leq C \bigl\Vert D^{m}u \bigr\Vert ^{\alpha}_{L^{r}(\mathbf{R}^{n})}\Vert u\Vert ^{1-\alpha}_{L^{q}(\mathbf{R}^{n})}, $$

*where* *C* *is a positive constant*, $0\leq\frac{j}{m}\leq \alpha\leq1$,

$$\frac{1}{p}=\frac{j}{n}+\alpha \biggl(\frac{1}{r}- \frac{m}{n} \biggr)+(1-\alpha)\frac{1}{q}. $$

### Lemma 2.3

*Suppose that* $E_{0}(x)\in H^{2}(\mathbf{R}^{2})$, $n_{0}(x)\in H^{1}(\mathbf{R}^{2})$, $\varphi_{0}(x)\in H^{1}(\mathbf{R}^{2})$. *Then we have*

$$\begin{aligned} \mathscr{F}(t)=\mathscr{F}(0), \end{aligned}$$

*where*

$$\begin{aligned} \mathscr{F}(t)=\Vert \nabla E\Vert ^{2}_{L^{2}}+H^{2} \Vert \Delta E\Vert ^{2}_{L^{2}}+ \int_{\mathbf{R}^{2}}n\vert E\vert ^{2}\,\mathrm{d} x+ \frac{1}{2}\Vert \nabla\varphi \Vert ^{2}_{L^{2}}+ \frac {1}{2}\Vert n\Vert ^{2}_{L^{2}}+ \frac{H^{2}}{2}\Vert \nabla n\Vert ^{2}_{L^{2}}. \end{aligned}$$

### Proof

Take the inner products of (6) and $-E_{t}$. Since

$$\begin{aligned} &\operatorname {Re}(iE_{t},-E_{t})=0, \qquad \operatorname {Re}(\Delta E,-E_{t})= \frac{1}{2}\frac{\mathrm{d}}{\mathrm{d}t}\Vert \nabla E\Vert ^{2}_{L^{2}}, \\ &\operatorname {Re}\bigl(-H^{2}\Delta^{2}E,-E_{t} \bigr)= \frac{H^{2}}{2}\frac{\mathrm{d}}{\mathrm{d}t}\Vert \Delta E\Vert ^{2}_{L^{2}}, \\ &\operatorname {Re}(-n E,-E_{t})=\frac{1}{2} \int_{\mathbf{R}^{2}}n\vert E\vert ^{2}_{t} \,\mathrm{d} x \\ &\phantom{\operatorname {Re}(-n E,-E_{t})}=\frac{1}{2}\frac{\mathrm{d}}{\mathrm{d} t} \int_{\mathbf{R}^{2}}n\vert E\vert ^{2}\,\mathrm{d} x- \frac{1}{2} \int_{\mathbf{R}^{2}}n_{t}\vert E\vert ^{2} \,\mathrm{d} x \\ &\phantom{\operatorname {Re}(-n E,-E_{t})}=\frac{1}{2}\frac{\mathrm{d}}{\mathrm{d} t} \int_{\mathbf{R}^{2}}n\vert E\vert ^{2}\,\mathrm{d} x- \frac{1}{2} \int_{\mathbf{R}^{2}}n_{t} \bigl(\varphi_{t}-n+H^{2} \Delta n \bigr)\,\mathrm{d} x \\ &\phantom{\operatorname {Re}(-n E,-E_{t})}=\frac{1}{2}\frac{\mathrm{d}}{\mathrm{d} t} \int_{\mathbf{R}^{2}}n\vert E\vert ^{2}\,\mathrm{d} x- \frac{1}{2} \int_{\mathbf{R}^{2}}n_{t}\varphi_{t}\,\mathrm{d} x+ \frac{1}{4}\frac{\mathrm{d}}{\mathrm{d} t}\Vert n\Vert ^{2}_{L^{2}}+ \frac{H^{2}}{4}\frac{\mathrm {d}}{\mathrm{d} t}\Vert \nabla n\Vert ^{2}_{L^{2}} \\ &\phantom{\operatorname {Re}(-n E,-E_{t})}=\frac{1}{2}\frac{\mathrm{d}}{\mathrm{d} t} \int_{\mathbf{R}^{2}}n\vert E\vert ^{2}\,\mathrm{d} x- \frac{1}{2} \int_{\mathbf{R}^{2}}\Delta\varphi\varphi_{t}\,\mathrm{d} x+ \frac{1}{4}\frac{\mathrm{d}}{\mathrm{d} t}\Vert n\Vert ^{2}_{L^{2}}+ \frac{H^{2}}{4}\frac{\mathrm {d}}{\mathrm{d} t}\Vert \nabla n\Vert ^{2}_{L^{2}} \\ &\phantom{\operatorname {Re}(-n E,-E_{t})}=\frac{1}{2}\frac{\mathrm{d}}{\mathrm{d}t} \int_{\mathbf{R}^{2}}n\vert E\vert ^{2}\,\mathrm{d} x+ \frac{1}{4}\frac{\mathrm{d}}{\mathrm{d} t}\Vert \nabla\varphi \Vert ^{2}_{L^{2}}+\frac{1}{4}\frac{\mathrm {d}}{\mathrm{d} t}\Vert n \Vert ^{2}_{L^{2}}+\frac{H^{2}}{4}\frac{\mathrm {d}}{\mathrm{d}t}\Vert \nabla n\Vert ^{2}_{L^{2}}, \end{aligned}$$

thus it follows that

10 $$\begin{aligned} &\frac{\mathrm{d}}{\mathrm{d}t} \biggl[\Vert \nabla E\Vert ^{2}_{L^{2}}+H^{2}\Vert \Delta E\Vert ^{2}_{L^{2}}+ \int_{\mathbf{R}^{2}}n\vert E\vert ^{2}\,\mathrm{d} x+ \frac{1}{2}\Vert \nabla\varphi \Vert ^{2}_{L^{2}}+ \frac {1}{2}\Vert n\Vert ^{2}_{L^{2}}+ \frac{H^{2}}{2}\Vert \nabla n\Vert ^{2}_{L^{2}} \biggr] \\ &\quad=0. \end{aligned}$$

Letting

$$\mathscr{F}(t)=\Vert \nabla E\Vert ^{2}_{L^{2}}+H^{2} \Vert \Delta E\Vert ^{2}_{L^{2}}+ \int_{\mathbf{R}^{2}}n\vert E\vert ^{2}\,\mathrm{d} x+ \frac{1}{2}\Vert \nabla\varphi \Vert ^{2}_{L^{2}}+ \frac {1}{2}\Vert n\Vert ^{2}_{L^{2}}+ \frac{H^{2}}{2}\Vert \nabla n\Vert ^{2}_{L^{2}}, $$

and noticing (10), we obtain

$$\begin{aligned} \mathscr{F}(t)=\mathscr{F}(0). \end{aligned}$$

 □

### Lemma 2.4

*Suppose that* $E_{0}(x)\in H^{2}(\mathbf{R}^{2})$, $n_{0}(x)\in H^{1}(\mathbf{R}^{2})$, $\varphi_{0}(x)\in H^{1}(\mathbf{R}^{2})$. *Then we have*

$$\sup_{0\leq t\leq T} \bigl(\Vert E\Vert _{H^{2}}+\Vert n \Vert _{H^{1}}+\Vert \varphi \Vert _{H^{1}} \bigr)\leq C. $$

### Proof

By Hölder’s inequality, Young’s inequality and Lemma 2.2, it follows that

$$\begin{aligned} \biggl\vert \int_{\mathbf{R}^{2}} n\vert E\vert ^{2}\,\mathrm{d}x \biggr\vert &\leq \Vert n\Vert _{L^{2}}\Vert E\Vert ^{2}_{L^{4}} \\ &\leq\frac{1}{4}\Vert n\Vert ^{2}_{L^{2}}+\Vert E \Vert ^{4}_{L^{4}} \\ &\leq\frac{1}{4}\Vert n\Vert ^{2}_{L^{2}}+C\Vert \Delta E\Vert _{L^{2}}\Vert E\Vert ^{3}_{L^{2}} \\ &\leq\frac{1}{4}\Vert n\Vert ^{2}_{L^{2}}+ \frac{H^{2}}{2}\Vert \Delta E\Vert ^{2}_{L^{2}}+C. \end{aligned}$$

From Lemma 2.3 we get

$$\Vert \nabla E\Vert ^{2}_{L^{2}}+\frac{H^{2}}{2}\Vert \Delta E\Vert ^{2}_{L^{2}}+\frac{1}{2}\Vert \nabla \varphi \Vert ^{2}_{L^{2}}+\frac{1}{4}\Vert n\Vert ^{2}_{L^{2}}+\frac{H^{2}}{2}\Vert \nabla n\Vert ^{2}_{L^{2}}\leq\mathscr{F}(0)+C. $$

Take the inner products of Eq. (8) and *φ*. It follows that

11 $$\begin{aligned} \bigl(\varphi_{t}-n+H^{2}\Delta n-\vert E \vert ^{2}, \varphi \bigr)=0 \end{aligned}$$

since

$$\begin{aligned} &(\varphi_{t}, \varphi )=\frac{1}{2}\frac{\mathrm{d}}{\mathrm {d}t}\Vert \varphi \Vert ^{2}_{L^{2}}, \\ &\bigl(n+\vert E\vert ^{2}, \varphi \bigr)\leq \bigl(\Vert n \Vert _{L^{2}}+\Vert E\Vert ^{2}_{L^{4}} \bigr) \Vert \varphi \Vert _{L^{2}}\leq\frac{1}{2}\Vert \varphi \Vert ^{2}_{L^{2}}+C, \end{aligned}$$

where

$$\begin{aligned} &\Vert E\Vert ^{2}_{L^{4}}\leq C \Vert \nabla E\Vert _{L^{2}}\Vert E\Vert _{L^{2}}\leq C. \\ &\bigl(H^{2}\Delta n, \varphi \bigr)=H^{2} (n, \Delta\varphi )=H^{2} (n, n_{t} )=\frac{H^{2}}{2}\frac{\mathrm{d}}{\mathrm {d}t} \Vert n\Vert ^{2}_{L^{2}}. \end{aligned}$$

Hence, from Eq. (11) we get

$$\begin{aligned} \frac{\mathrm{d}}{\mathrm{d}t} \bigl(\Vert \varphi \Vert ^{2}_{L^{2}}+H^{2} \Vert n\Vert ^{2}_{L^{2}} \bigr)\leq \Vert \varphi \Vert ^{2}_{L^{2}}+C. \end{aligned}$$

Using Gronwall’s inequality, we obtain that

$$\begin{aligned} \sup_{0\leq t\leq T} \bigl(\Vert \varphi \Vert ^{2}_{L^{2}}+H^{2} \Vert n\Vert ^{2}_{L^{2}} \bigr)\leq C. \end{aligned}$$

We thus get Lemma 2.4. □

### Lemma 2.5

*Suppose that* $E_{0}(x)\in H^{4}(\mathbf{R}^{2})$, $n_{0}(x)\in H^{2}(\mathbf{R}^{2})$, $\varphi_{0}(x)\in H^{2}(\mathbf{R}^{2})$. *Then we have*

$$\sup_{0\leq t \leq T} \bigl(\Vert E\Vert _{H^{4}}+\Vert n \Vert _{H^{2}}+\Vert \varphi \Vert _{H^{2}}+\Vert E_{t}\Vert _{L^{2}}+\Vert n_{t}\Vert _{L^{2}}+\Vert \varphi_{t}\Vert _{L^{2}} \bigr)\leq C. $$

### Proof

Differentiating (6) with respect to *t*, then taking the inner products of the resulting equation and $E_{t}$, we have

12 $$\begin{aligned} \bigl(iE_{tt}+\Delta E_{t}-H^{2} \Delta^{2}E_{t}- (nE )_{t}, E_{t} \bigr)=0 \end{aligned}$$

since

$$\begin{aligned} &\operatorname {Im}(iE_{tt},E_{t})=\frac{1}{2}\frac{\mathrm{d}}{\mathrm{d}t} \Vert E_{t}\Vert ^{2}_{L^{2}}, \qquad \operatorname {Im}\bigl(\Delta E_{t}-H^{2}\Delta^{2}E_{t}-nE_{t},E_{t} \bigr)=0, \\ &\bigl\vert \operatorname {Im}(-n_{t}E,E_{t}) \bigr\vert \leq C\Vert E\Vert _{L^{\infty}} \Vert n_{t}\Vert _{L^{2}}\Vert E_{t}\Vert _{L^{2}}\leq C \bigl(\Vert E_{t} \Vert ^{2}_{L^{2}}+\Vert n_{t}\Vert ^{2}_{L^{2}} \bigr). \end{aligned}$$

By Lemma 2.2, it follows that

$$\begin{aligned} \Vert E\Vert _{L^{\infty}}\leq C\Vert \Delta E\Vert ^{\frac{1}{2}}_{L^{2}}\Vert E\Vert ^{\frac{1}{2}}_{L^{2}}; \end{aligned}$$

thus from Eq. (12) we get

13 $$ \frac{\mathrm{d}}{\mathrm{d}t}\Vert E_{t}\Vert ^{2}_{L^{2}}\leq C \bigl(\Vert E_{t}\Vert ^{2}_{L^{2}}+\Vert n_{t}\Vert ^{2}_{L^{2}} \bigr). $$

Differentiating Eq. (7) with respect to *t*, then taking the inner products of the resulting equation and $n_{t}$, we have

14 $$\begin{aligned} (n_{tt}-\Delta\varphi_{t}, n_{t} )=0 \end{aligned}$$

since

$$\begin{aligned} &(n_{tt}, n_{t} )=\frac{1}{2}\frac{\mathrm{d}}{\mathrm{d}t}\Vert n_{t}\Vert ^{2}_{L^{2}}, \\ &(-\Delta\varphi_{t}, n_{t} )= \bigl(-\Delta n+H^{2}\Delta^{2} n-\Delta \vert E\vert ^{2}, n_{t} \bigr) \\ & = \frac{1}{2}\frac{\mathrm{d}}{\mathrm{d}t}\Vert \nabla n\Vert ^{2}_{L^{2}}+\frac{H^{2}}{2}\frac{\mathrm{d}}{\mathrm{d}t}\Vert \Delta n\Vert ^{2}_{L^{2}}- \bigl(\Delta \vert E\vert ^{2}, n_{t} \bigr). \end{aligned}$$

Noting that

$$\begin{aligned} \bigl\vert \bigl(\Delta \vert E\vert ^{2}, n_{t} \bigr) \bigr\vert \leq C \Vert E\Vert _{L^{\infty}} \Vert \Delta E\Vert _{L^{2}}\Vert n_{t}\Vert _{L^{2}}\leq C \bigl( \Vert n_{t}\Vert ^{2}_{L^{2}}+1 \bigr), \end{aligned}$$

from Eq. (14) we get

15 $$\begin{aligned} \frac{\mathrm{d}}{\mathrm{d}t} \bigl[\Vert n_{t}\Vert ^{2}_{L^{2}}+\Vert \nabla n\Vert ^{2}_{L^{2}}+H^{2} \Vert \Delta n\Vert ^{2}_{L^{2}} \bigr]\leq C \bigl(\Vert n_{t}\Vert ^{2}_{L^{2}}+1 \bigr). \end{aligned}$$

From Eq. (13) and (15) we get

$$\begin{aligned} \frac{\mathrm{d}}{\mathrm{d}t} \bigl[\Vert E_{t}\Vert ^{2}_{L^{2}}+ \Vert n_{t}\Vert ^{2}_{L^{2}}+\Vert \nabla n \Vert ^{2}_{L^{2}}+H^{2}\Vert \Delta n\Vert ^{2}_{L^{2}} \bigr]\leq C \bigl(\Vert E_{t}\Vert ^{2}_{L^{2}}+\Vert n_{t}\Vert ^{2}_{L^{2}}+1 \bigr). \end{aligned}$$

By Gronwall’s inequality, it follows that

16 $$\begin{aligned} \sup_{0\leq t\leq T} \bigl[\Vert E_{t} \Vert ^{2}_{L^{2}}+\Vert n_{t}\Vert ^{2}_{L^{2}}+\Vert \nabla n\Vert ^{2}_{L^{2}}+H^{2} \Vert \Delta n\Vert ^{2}_{L^{2}} \bigr]\leq C. \end{aligned}$$

Take the inner products of Eq. (8) and $\varphi_{t}$. It follows that

17 $$\begin{aligned} \bigl(\varphi_{t}-n+H^{2}\Delta n-\vert E \vert ^{2}, \varphi _{t} \bigr)=0 \end{aligned}$$

since

$$\begin{aligned} &(\varphi_{t}, \varphi_{t} )=\Vert \varphi_{t} \Vert ^{2}_{L^{2}}, \\ &\bigl(-n+H^{2}\Delta n-\vert E\vert ^{2}, \varphi_{t} \bigr)\leq \bigl(\Vert n\Vert _{L^{2}}+H^{2} \Vert \Delta n\Vert _{L^{2}}+\Vert E\Vert ^{2}_{L^{4}} \bigr)\Vert \varphi_{t}\Vert _{L^{2}} \\ & \phantom{\bigl(-n+H^{2}\Delta n-\vert E\vert ^{2}, \varphi_{t} \bigr)}\leq C+\frac{1}{2}\Vert \varphi_{t}\Vert ^{2}_{L^{2}}. \end{aligned}$$

From Eq. (17) we get

$$\begin{aligned} \Vert \varphi_{t}\Vert ^{2}_{L^{2}}\leq C. \end{aligned}$$

Take the inner products of Eq. (6) and Δ*E*. It follows that

18 $$\begin{aligned} \bigl(iE_{t}+\Delta E-H^{2} \Delta^{2}E-nE, \Delta E \bigr)=0 \end{aligned}$$

since

$$\begin{aligned} &(iE_{t}-nE, \Delta E )\leq \bigl(\Vert E_{t}\Vert _{L^{2}}+\Vert E\Vert _{L^{\infty}} \Vert n\Vert _{L^{2}} \bigr)\Vert \Delta E\Vert _{L^{2}}\leq C, \\ &\bigl(\Delta E-H^{2}\Delta^{2}E, \Delta E \bigr)=\Vert \Delta E\Vert ^{2}_{L^{2}}+H^{2} \bigl\Vert \nabla^{3} E \bigr\Vert ^{2}_{L^{2}}. \end{aligned}$$

From Eq. (18) we get

$$\begin{aligned} \bigl\Vert \nabla^{3} E \bigr\Vert ^{2}_{L^{2}} \leq C. \end{aligned}$$

From (6) we obtain

$$\begin{aligned} H^{2} \bigl\Vert \Delta^{2}E \bigr\Vert _{L^{2}} \leq \Vert E_{t}\Vert _{L^{2}}+\Vert \Delta E\Vert _{L^{2}}+\Vert nE\Vert _{L^{2}}\leq C, \end{aligned}$$

where

$$\begin{aligned} \Vert nE\Vert _{L^{2}}\leq \Vert n\Vert _{L^{4}}\Vert E \Vert _{L^{4}}\leq C \Vert \nabla n\Vert ^{\frac {1}{2}}_{L^{2}} \Vert n\Vert ^{\frac{1}{2}}_{L^{2}}\Vert \nabla E\Vert ^{\frac{1}{2}}_{L^{2}}\Vert E\Vert ^{\frac {1}{2}}_{L^{2}}\leq C. \end{aligned}$$

From (7) we obtain

$$\begin{aligned} \Vert \Delta\varphi \Vert _{L^{2}}=\Vert n_{t}\Vert _{L^{2}}\leq C. \end{aligned}$$

We thus get Lemma 2.5. □

### Lemma 2.6

*Suppose that* $f_{1},f_{2}\in H^{s}(\Omega)$, $\Omega\subseteq\mathbf{R}^{n}$. *Then we have*

$$\bigl\Vert D^{s}(f_{1}\cdot f_{2}) \bigr\Vert _{L^{2}}\leq C_{s} \bigl(\Vert f_{1}\Vert _{L^{2}} \bigl\Vert D^{s}f_{2} \bigr\Vert _{L^{2}}+ \bigl\Vert D^{s}f_{1} \bigr\Vert _{L^{2}}\Vert f_{2}\Vert _{L^{2}} \bigr), $$

*where the constant* $C_{s}$ *is independent of* $f_{1}$ *and* $f_{2}$.

### Lemma 2.7

*Suppose that* $E_{0}(x)\in H^{m+4}(\mathbf{R}^{2})$, $n_{0}(x)\in H^{m+2}(\mathbf{R}^{2})$, $\varphi_{0}(x)\in H^{m+2}(\mathbf{R}^{2})$, $m\geq0$. *Then we have*

$$\begin{aligned} &\sup_{0\leq t\leq T} \bigl( \bigl\Vert E(x,t) \bigr\Vert _{H^{m+4}}+ \bigl\Vert n(x,t) \bigr\Vert _{H^{m+2}}+ \bigl\Vert \varphi(x,t) \bigr\Vert _{H^{m+2}} \bigr)\leq C \\ &\sup_{0\leq t\leq T} \bigl( \bigl\Vert E_{t}(x,t) \bigr\Vert _{H^{m}}+ \bigl\Vert n_{t}(x,t) \bigr\Vert _{H^{m}}+\Vert \varphi_{t}\Vert _{H^{m}} \bigr)\leq C. \end{aligned}$$

### Proof

Lemma 2.7 is true when $m=0$ (Lemma 2.5). Suppose that Lemma 2.7 is true when $m=k, (k\geq0)$. Take the inner products of (8) and $(-1)^{k+1}\Delta ^{k+3}\varphi$. It follows that

19 $$\begin{aligned} \bigl(\varphi_{t}-n+H^{2}\Delta n-\vert E \vert ^{2}, (-1)^{k+1}\Delta^{k+3}\varphi \bigr)=0 \end{aligned}$$

since

$$\begin{aligned} &\bigl(\varphi_{t},(-1)^{k+1}\Delta^{k+3}\varphi \bigr)=\frac{1}{2}\frac {\mathrm{d}}{\mathrm{d}t} \bigl\Vert \nabla^{k+3} \varphi \bigr\Vert ^{2}_{L^{2}}, \\ &\bigl(-n,(-1)^{k+1}\Delta^{k+3}\varphi \bigr)= \bigl(-n,(-1)^{k+1}\Delta^{k+2} n_{t} \bigr)= \frac{1}{2}\frac{\mathrm{d}}{\mathrm{d}t} \bigl\Vert \nabla^{k+2} n \bigr\Vert ^{2}_{L^{2}}, \\ &\bigl(H^{2}\Delta n,(-1)^{k+1}\Delta^{k+3}\varphi \bigr)=H^{2} \bigl(\Delta n,(-1)^{k+1}\Delta^{k+2} n_{t} \bigr)=\frac{H^{2}}{2}\frac{\mathrm{d}}{\mathrm{d}t} \bigl\Vert \nabla^{k+3} n \bigr\Vert ^{2}_{L^{2}}, \\ &\bigl\vert \bigl(-\vert E\vert ^{2},(-1)^{k+1}\Delta ^{k+3}\varphi \bigr) \bigr\vert = \bigl\vert \bigl( \nabla^{k+3} \vert E\vert ^{2},\nabla^{k+3} \varphi \bigr) \bigr\vert \leq \bigl\Vert \nabla^{k+3} \vert E \vert ^{2} \bigr\Vert _{L^{2}} \bigl\Vert \nabla^{k+3} \varphi \bigr\Vert _{L^{2}} \\ &\phantom{\bigl\vert \bigl(-\vert E\vert ^{2},(-1)^{k+1}\Delta ^{k+3}\varphi \bigr) \bigr\vert }\leq C\Vert E\Vert _{L^{2}} \bigl\Vert \nabla^{k+3} E \bigr\Vert _{L^{2}} \bigl\Vert \nabla^{k+3}\varphi \bigr\Vert _{L^{2}} \\ &\phantom{\bigl\vert \bigl(-\vert E\vert ^{2},(-1)^{k+1}\Delta ^{k+3}\varphi \bigr) \bigr\vert }\leq C \bigl( \bigl\Vert \nabla^{k+3}\varphi \bigr\Vert ^{2}_{L^{2}}+1 \bigr), \end{aligned}$$

thus from Eq. (19) it follows that

20 $$\begin{aligned} \frac{\mathrm{d}}{\mathrm{d}t} \bigl( \bigl\Vert \nabla^{k+3} \varphi \bigr\Vert ^{2}_{L^{2}}+ \bigl\Vert \nabla^{k+2} n \bigr\Vert ^{2}_{L^{2}}+H^{2} \bigl\Vert \nabla^{k+3} n \bigr\Vert ^{2}_{L^{2}} \bigr)\leq C \bigl( \bigl\Vert \nabla^{k+3}\varphi \bigr\Vert ^{2}_{L^{2}}+1 \bigr). \end{aligned}$$

By using Gronwall’s inequality, we have

$$\sup_{0\leq t\leq T} \bigl( \bigl\Vert \nabla^{k+3}\varphi \bigr\Vert ^{2}_{L^{2}}+ \bigl\Vert \nabla^{k+2} n \bigr\Vert ^{2}_{L^{2}}+ \bigl\Vert \nabla^{k+3} n \bigr\Vert ^{2}_{L^{2}} \bigr)\leq C. $$

From (7) and (8) we get

$$\begin{aligned} & \bigl\Vert \nabla^{k+1}n_{t} \bigr\Vert _{L^{2}}= \bigl\Vert \nabla ^{k+3}\varphi \bigr\Vert _{L^{2}}\leq C, \\ & \bigl\Vert \nabla^{k+1}\varphi_{t} \bigr\Vert _{L^{2}}\leq C \bigl( \bigl\Vert \nabla^{k+1}n \bigr\Vert _{L^{2}}+ \bigl\Vert \nabla^{k+3} n \bigr\Vert _{L^{2}}+ \Vert E\Vert _{L^{2}} \bigl\Vert \nabla ^{k+1}E \bigr\Vert _{L^{2}} \bigr)\leq C. \end{aligned}$$

Differentiating (6) with respect to *t*, then taking the inner products of the resulting equation and $(-1)^{k+1}\Delta ^{k+1}E_{t}$, we obtain

21 $$\begin{aligned} \bigl(iE_{tt}+\Delta E_{t}-H^{2} \Delta^{2}E_{t}- (nE )_{t}, (-1)^{k+1} \Delta^{k+1}E_{t} \bigr)=0. \end{aligned}$$

Since

$$\begin{aligned} &\operatorname {Im}\bigl(iE_{tt},(-1)^{k+1}\Delta^{k+1}E_{t} \bigr)=\frac{1}{2}\frac {\mathrm{d}}{\mathrm{d}t} \bigl\Vert \nabla^{k+1}E_{t} \bigr\Vert ^{2}_{L^{2}}, \\ &\operatorname {Im}\bigl(\Delta E_{t}-H^{2}\Delta^{2}E_{t},(-1)^{k+1} \Delta^{k+1}E_{t} \bigr)=0, \\ &\bigl\vert \operatorname {Im}\bigl(-n_{t}E,(-1)^{k+1} \Delta^{k+1}E_{t} \bigr) \bigr\vert \leq \bigl\vert \bigl( \nabla^{k+1}(n_{t}E),\nabla^{k+1}E_{t} \bigr) \bigr\vert \\ &\phantom{\bigl\vert \operatorname {Im}\bigl(-n_{t}E,(-1)^{k+1} \Delta^{k+1}E_{t} \bigr) \bigr\vert }\leq C \bigl( \bigl\Vert \nabla^{k+1}n_{t} \bigr\Vert _{L^{2}}\Vert E\Vert _{L^{2}}+ \bigl\Vert \nabla^{k+1}E \bigr\Vert _{L^{2}}\Vert n_{t}\Vert _{L^{2}} \bigr) \bigl\Vert \nabla^{k+1}E_{t} \bigr\Vert _{L^{2}} \\ &\phantom{\bigl\vert \operatorname {Im}\bigl(-n_{t}E,(-1)^{k+1} \Delta^{k+1}E_{t} \bigr) \bigr\vert }\leq C \bigl( \bigl\Vert \nabla^{k+1}E_{t} \bigr\Vert ^{2}_{L^{2}}+1 \bigr), \\ &\bigl\vert \operatorname {Im}\bigl(-nE_{t},(-1)^{k+1} \Delta^{k+1}E_{t} \bigr) \bigr\vert \leq \bigl\vert \bigl( \nabla^{k+1}(nE_{t}),\nabla^{k+1}E_{t} \bigr) \bigr\vert \\ &\phantom{\bigl\vert \operatorname {Im}\bigl(-nE_{t},(-1)^{k+1} \Delta^{k+1}E_{t} \bigr) \bigr\vert }\leq C \bigl( \bigl\Vert \nabla^{k+1}n \bigr\Vert _{L^{2}} \Vert E_{t}\Vert _{L^{2}}+ \bigl\Vert \nabla^{k+1}E_{t} \bigr\Vert _{L^{2}}\Vert n\Vert _{L^{2}} \bigr) \bigl\Vert \nabla^{k+1}E_{t} \bigr\Vert _{L^{2}} \\ &\phantom{\bigl\vert \operatorname {Im}\bigl(-nE_{t},(-1)^{k+1} \Delta^{k+1}E_{t} \bigr) \bigr\vert }\leq C \bigl( \bigl\Vert \nabla^{k+1}E_{t} \bigr\Vert ^{2}_{L^{2}}+1 \bigr), \end{aligned}$$

thus from Eq. (21) we get

22 $$ \frac{\mathrm{d}}{\mathrm{d}t} \bigl\Vert \nabla^{k+1}E_{t} \bigr\Vert ^{2}_{L^{2}}\leq C \bigl( \bigl\Vert \nabla^{k+1}E_{t} \bigr\Vert ^{2}_{L^{2}}+1 \bigr). $$

By using Gronwall’s inequality, we get

$$\sup_{0\leq t \leq T} \bigl\Vert \nabla^{k+1}E_{t} \bigr\Vert ^{2}_{L^{2}}\leq C. $$

From (6) we obtain

$$\begin{aligned} \bigl\Vert \nabla^{k+5}E \bigr\Vert _{L^{2}}\leq C \bigl( \bigl\Vert \nabla ^{k+1}E_{t} \bigr\Vert _{L^{2}}+ \bigl\Vert \nabla^{k+3} E \bigr\Vert _{L^{2}}+ \bigl\Vert \nabla^{k+1}n \bigr\Vert _{L^{2}}\Vert E\Vert _{L^{2}}+\Vert n\Vert _{L^{2}} \bigl\Vert \nabla^{k+1}E \bigr\Vert _{L^{2}} \bigr)\leq C. \end{aligned}$$

Hence

$$\begin{aligned} &\sup_{0\leq t\leq T} \bigl( \bigl\Vert E(x,t) \bigr\Vert _{H^{k+5}}+ \bigl\Vert n(x,t) \bigr\Vert _{H^{k+3}}+ \bigl\Vert \varphi(x,t) \bigr\Vert _{H^{k+3}} \bigr)\leq C, \\ &\sup_{0\leq t\leq T} \bigl( \bigl\Vert E_{t}(x,t) \bigr\Vert _{H^{k+1}}+ \bigl\Vert n_{t}(x,t) \bigr\Vert _{H^{k+1}}+\Vert \varphi_{t}\Vert _{H^{k+1}} \bigr)\leq C. \end{aligned}$$

This means Lemma 2.7 is true when $m=k+1$. Thus Lemma 2.7 is proved completely. □

## Existence and uniqueness of solution

Now, with these lemmas, we are able to prove Theorem 1.1. First we obtain the existence and uniqueness of the global generalized solution of problem (6)-(9).

### Definition 3.1

The functions $E\in L^{\infty}(0, T; H^{4})\cap W^{1,\infty}(0, T; L^{2})$, $n\in L^{\infty}(0, T; H^{2})\cap W^{1,\infty}(0, T; L^{2})$ and $\varphi \in L^{\infty}(0, T; H^{2})\cap W^{1,\infty}(0, T; L^{2})$ are called a generalized solution of problem (6)-(9) if for any $\omega\in L^{2}$ they satisfy the integral equality

$$\begin{aligned} & (iE_{jt}, \omega )+ (\Delta E_{j}, \omega )=H^{2} \bigl(\Delta^{2}E_{j}, \omega \bigr)+ (nE_{j},\omega ),\quad j=1,2,\ldots,N, \\ & (n_{t},\omega )= (\Delta\varphi,\omega ), \\ & (\varphi_{t},\omega )+H^{2} (\Delta n,\omega )= (n,\omega )+ \bigl(\vert E\vert ^{2},\omega \bigr) \end{aligned}$$

with initial data

$$E(x,0)=E_{0}(x),\qquad n(x,0)=n_{0}(x),\qquad \varphi(x,0)= \varphi_{0}(x). $$

Now, one can estimate the following theorem.

### Theorem 3.1

*Suppose that* $E_{0}(x)\in H^{l+4}(\mathbf{R}^{2})$, $n_{0}(x)\in H^{l+2}(\mathbf{R}^{2})$, $\varphi_{0}(x)\in H^{l+2}(\mathbf{R}^{2})$, $l\geq0$. *Then there exists a global smooth solution of the initial value problem* (6)-(9).

$$\begin{aligned} &E(x,t)\in L^{\infty}\bigl(0,T;H^{l+4} \bigl( \mathbf{R}^{2} \bigr) \bigr), \qquad E_{t}(x,t)\in L^{\infty}\bigl(0,T;H^{l} \bigl(\mathbf{R}^{2} \bigr) \bigr), \\ &n(x,t)\in L^{\infty}\bigl(0,T;H^{l+2} \bigl( \mathbf{R}^{2} \bigr) \bigr), \qquad n_{t}(x,t)\in L^{\infty}\bigl(0,T;H^{l} \bigl(\mathbf{R}^{2} \bigr) \bigr), \\ &\varphi(x,t)\in L^{\infty}\bigl(0,T;H^{l+2} \bigl( \mathbf{R}^{2} \bigr) \bigr), \qquad \varphi_{t}(x,t)\in L^{\infty}\bigl(0,T;H^{l} \bigl(\mathbf{R}^{2} \bigr) \bigr). \end{aligned}$$

### Proof

By using the Galerkin method, choose the basic periodic functions $\{\omega_{\kappa}(x)\}$ as follows:

$$-\Delta\omega_{\kappa}(x)=\lambda_{\kappa}\omega_{\kappa}(x),\quad \omega_{\kappa}(x)\in H^{2}(\Omega), \kappa=1,\ldots, l. $$

The approximate solution of problem (6)-(9) can be written as

$$\begin{aligned} &E^{l}(x,t)=\sum^{l}_{\kappa=1} \alpha^{l}_{\kappa}(t)\omega_{\kappa}(x),\qquad n^{l}(x,t)=\sum^{l}_{\kappa=1} \beta^{l}_{\kappa}(t)\omega_{\kappa}(x), \\ &\varphi^{l}(x,t)=\sum^{l}_{\kappa=1} \gamma^{l}_{\kappa}(t)\omega_{\kappa}(x), \end{aligned}$$

where

$$\begin{aligned} E^{l}(x,t)= \bigl(E^{l}_{1}, E^{l}_{2}, \ldots, E^{l}_{N} \bigr), \qquad \alpha^{l}_{\kappa}(t)= \bigl(\alpha^{l}_{\kappa1}, \alpha^{l}_{\kappa 2}, \ldots, \alpha^{l}_{\kappa N} \bigr). \end{aligned}$$

Ω is a two-dimensional cube with 2*D* in each direction, that is, $\overline{\Omega}=\{x=(x_{1}, x_{2})\vert \vert x_{j}\vert \leq2D, j=1, 2\}$. According to Galerkin’s method, these undetermined coefficients $\alpha^{l}_{\kappa}(t)$, $\beta^{l}_{\kappa}(t)$ and $\gamma^{l}_{\kappa }(t)$ need to satisfy the following initial value problem of the system of ordinary differential equations:

23 $$\begin{aligned} & \bigl(iE^{l}_{jt}, \omega_{\kappa}\bigr)+ \bigl(\Delta E^{l}_{j}, \omega_{\kappa}\bigr)=H^{2} \bigl(\Delta^{2}E^{l}_{j}, \omega_{\kappa}\bigr)+ \bigl(n^{l}E^{l}_{j}, \omega_{\kappa}\bigr),\quad j=1,2,\ldots,N, \end{aligned}$$

24 $$\begin{aligned} & \bigl(n^{l}_{t},\omega_{\kappa}\bigr)= \bigl(\Delta\varphi ^{l},\omega_{\kappa}\bigr), \end{aligned}$$

25 $$\begin{aligned} & \bigl(\varphi^{l}_{t},\omega_{\kappa}\bigr)+H^{2} \bigl(\Delta n^{l},\omega_{\kappa}\bigr)= \bigl(n^{l},\omega_{\kappa}\bigr)+ \bigl( \bigl\vert E^{l} \bigr\vert ^{2},\omega_{\kappa}\bigr) \end{aligned}$$

with initial data

26 $$\begin{aligned} E^{l}(x,0)=E^{l}_{0}(x),\qquad n^{l}(x,0)=n^{l}_{0}(x), \qquad \varphi^{l}(x,0)= \varphi^{l}_{0}(x), \end{aligned}$$

where

$$E^{l}_{0}(x)\xrightarrow{H^{4}} E_{0}(x),\qquad n^{l}_{0}(x)\xrightarrow{H^{2}}n_{0}(x),\qquad \varphi^{l}_{0}(x)\xrightarrow{H^{2}} \varphi_{0}(x),\quad l\to\infty. $$

Similarly to the proof of Lemmas 2.1-2.5, for the solution $E^{l}(x,t)$, $n^{l}(x,t)$, $\varphi^{l}(x,t)$ of problem (23)-(26), we can establish the following estimates:

$$\begin{aligned} \sup_{0\leq t \leq T} \bigl( \bigl\Vert E^{l} \bigr\Vert _{H^{4}}+ \bigl\Vert n^{l} \bigr\Vert _{H^{2}}+ \bigl\Vert \varphi^{l} \bigr\Vert _{H^{2}}+ \bigl\Vert E^{l}_{t} \bigr\Vert _{L^{2}}+ \bigl\Vert n^{l}_{t} \bigr\Vert _{L^{2}}+ \bigl\Vert \varphi^{l}_{t} \bigr\Vert _{L^{2}} \bigr)\leq C, \end{aligned}$$

where the constants *C* are independent of *l* and *D*. By compact argument, some subsequence of $(E^{l}, n^{l}, \varphi^{l} )$, also labeled by *l*, has a weak limit $(E, n, \varphi )$. More precisely

$$\begin{aligned} & E^{l}(x,t)\to E(x,t) \quad\text{in } L^{\infty}\bigl(0,T; H^{4} \bigr) \text{ weakly star}, \\ & n^{l}(x,t)\to n(x,t)\quad \text{in } L^{\infty}\bigl(0,T; H^{2} \bigr) \text{ weakly star}, \\ & \varphi^{l}(x,t)\to\varphi(x,t) \quad\text{in } L^{\infty}\bigl(0,T;H^{2} \bigr) \text{ weakly star} \end{aligned}$$

and

$$\begin{aligned} & E^{l}_{t}\rightarrow E_{t} \quad\text{in } L^{\infty} \bigl(0,T; L^{2} \bigr) \text{ weakly star}, \\ & n^{l}_{t}\rightarrow n_{t} \quad\text{in } L^{\infty} \bigl(0,T; L^{2} \bigr) \text{ weakly star}, \\ & \varphi^{l}_{t}\rightarrow\varphi_{t}\quad \text{in } L^{\infty} \bigl(0,T; L^{2} \bigr) \text{ weakly star}. \end{aligned}$$

By using Guo and Shen’s method [15], one can prove the existence of a local solution for the periodic initial problem (6)-(9). Similarly to Zhou and Guo’s proof [16], letting $D\rightarrow\infty$, the existence of a local solution for the initial value problem (6)-(9) can be obtained. By the continuation extension principle, from the conditions of the theorem and *a priori* estimates in Section 2, we can get the existence of a global generalized solution for problem (6)-(9). By Lemma 2.7 and the Sobolev imbedding theorem, Theorem 3.1 is proved. □

Next, we prove the uniqueness of a solution for problem (6)-(9).

### Theorem 3.2

*Suppose that* $E_{0}(x)\in H^{l+4}(\mathbf{R}^{2})$, $n_{0}(x)\in H^{l+2}(\mathbf{R}^{2})$, $\varphi_{0}(x)\in H^{l+2}(\mathbf{R}^{2})$, $l\geq0$. *Then the global solution of the initial value problem* (6)-(9) *is unique*.

### Proof

Suppose that there are two solutions $E_{1},n_{1},\varphi_{1}$ and $E_{2},n_{2},\varphi_{2}$. Let

$$\begin{aligned} E=E_{1}-E_{2},\qquad n=n_{1}-n_{2},\qquad \varphi= \varphi_{1}-\varphi_{2}. \end{aligned}$$

From (6)-(9) we get

27 $$\begin{aligned} &iE_{t}+\Delta E-H^{2}\Delta^{2}E-n_{1}E_{1}+n_{2}E_{2}=0, \end{aligned}$$

28 $$\begin{aligned} &n_{t}-\Delta\varphi=0, \end{aligned}$$

29 $$\begin{aligned} &\varphi_{t}-n+H^{2}\Delta n-\vert E_{1}\vert ^{2}+\vert E_{2}\vert ^{2}=0, \end{aligned}$$

with initial data

30 $$\begin{aligned} &E\vert_{t=0}=0,\qquad n\vert_{t=0}=0,\qquad \varphi \vert_{t=0}=0,\qquad x\in\mathbf{R}^{2}. \end{aligned}$$

Take the inner product of (27) and *E*. Since

$$\begin{aligned} &\operatorname {Im}(iE_{t},E)=\frac{1}{2}\frac{\mathrm{d}}{\mathrm{d}t}\Vert E\Vert ^{2}_{L^{2}}, \\ &\operatorname {Im}\bigl(\Delta E-H^{2} \Delta^{2}E,E \bigr)=0, \\ &\bigl\vert \operatorname {Im}(n_{1}E_{1}-n_{2}E_{2},E ) \bigr\vert \leq \bigl\vert (nE_{1}+n_{2}E,E ) \bigr\vert \\ &\phantom{\bigl\vert \operatorname {Im}(n_{1}E_{1}-n_{2}E_{2},E ) \bigr\vert }\leq C \bigl(\Vert E_{1}\Vert _{L^{\infty}} \Vert n\Vert _{L^{2}}+\Vert n_{2}\Vert _{L^{\infty}} \Vert E\Vert _{L^{2}} \bigr)\Vert E\Vert _{L^{2}} \\ &\phantom{\bigl\vert \operatorname {Im}(n_{1}E_{1}-n_{2}E_{2},E ) \bigr\vert }\leq C \bigl(\Vert n\Vert ^{2}_{L^{2}}+\Vert E\Vert ^{2}_{L^{2}} \bigr), \end{aligned}$$

thus we obtain

31 $$ \frac{\mathrm{d}}{\mathrm{d}t}\Vert E\Vert ^{2}_{L^{2}} \leq C \bigl(\Vert n\Vert ^{2}_{L^{2}}+\Vert E\Vert ^{2}_{L^{2}} \bigr). $$

Take the inner product of (29) and *φ*. Since

$$\begin{aligned} &(\varphi_{t},\varphi)=\frac{1}{2}\frac{\mathrm{d}}{\mathrm{d}t}\Vert \varphi \Vert ^{2}_{L^{2}}, \qquad \bigl\vert (-n,\varphi) \bigr\vert \leq C \bigl(\Vert n\Vert ^{2}_{L^{2}}+\Vert \varphi \Vert ^{2}_{L^{2}} \bigr), \\ &\bigl(H^{2}\Delta n,\varphi \bigr)= \bigl(H^{2}n,\Delta \varphi \bigr)= \bigl(H^{2}n,n_{t} \bigr)=\frac{H^{2}}{2} \frac{\mathrm {d}}{\mathrm{d}t}\Vert n\Vert ^{2}_{L^{2}}, \\ &\bigl\vert \bigl(-\vert E_{1}\vert ^{2}+\vert E_{2}\vert ^{2},\varphi \bigr) \bigr\vert = \bigl\vert \bigl( (E_{1}-E_{2} )\overline{E_{1}}+E_{2}( \overline{E_{1}}-\overline{E_{2}}),\varphi \bigr) \bigr\vert \\ &\phantom{\bigl\vert \bigl(-\vert E_{1}\vert ^{2}+\vert E_{2}\vert ^{2},\varphi \bigr) \bigr\vert }\leq C (\Vert E_{1}\Vert_{L^{\infty}}\Vert E\Vert_{L^{2}} \Vert+\Vert E_{2}\Vert_{L^{\infty}}\Vert E\Vert_{L^{2}} )\Vert \varphi\Vert _{L^{2}} \\ &\phantom{\bigl\vert \bigl(-\vert E_{1}\vert ^{2}+\vert E_{2}\vert ^{2},\varphi \bigr) \bigr\vert }\leq C \bigl(\Vert E\Vert^{2}_{L^{2}}+\Vert\varphi \Vert^{2}_{L^{2}} \bigr), \end{aligned}$$

thus we get

32 $$ \frac{\mathrm{d}}{\mathrm{d}t} \bigl(\Vert \varphi \Vert ^{2}_{L^{2}}+H^{2}\Vert n\Vert ^{2}_{L^{2}} \bigr)\leq C \bigl(\Vert E\Vert ^{2}_{L^{2}}+\Vert n\Vert ^{2}_{L^{2}}+ \Vert \varphi \Vert ^{2}_{L^{2}} \bigr). $$

Hence from (31) and (32) we get

33 $$ \frac{\mathrm{d}}{\mathrm{d}t} \bigl(\Vert E\Vert ^{2}_{L^{2}}+ \Vert n\Vert ^{2}_{L^{2}}+\Vert \varphi \Vert ^{2}_{L^{2}} \bigr)\leq C \bigl(\Vert E\Vert ^{2}_{L^{2}}+\Vert n\Vert ^{2}_{L^{2}}+ \Vert \varphi \Vert ^{2}_{L^{2}} \bigr). $$

By using Gronwall’s inequality and noticing (30), we arrive at

$$E\equiv0, \qquad n\equiv0,\qquad \varphi\equiv0. $$

Theorem 3.2 is proved. This completes the proof of Theorem 1.1. □

## Results and discussion

One can regard (3)-(4) as the Langmuir turbulence parameterized by *H* and study the asymptotic behavior of systems (3)-(4) when *H* goes to zero.

## Conclusions

By *a priori* integral estimates and the Galerkin method, we have the following conclusion.

Suppose that $E_{0}(x)\in H^{l+4}(\mathbf{R}^{2})$, $n_{0}(x)\in H^{l+2}(\mathbf{R}^{2})$, $n_{1}(x)\in H^{l}(\mathbf{R}^{2})$, $l\geq0$. Then there exists a unique global smooth solution of the initial value problem (3)-(5).

$$\begin{aligned} &E(x,t)\in L^{\infty}\bigl(0,T;H^{l+4} \bigl( \mathbf{R}^{2} \bigr) \bigr),\qquad E_{t}(x,t)\in L^{\infty}\bigl(0,T;H^{l} \bigl(\mathbf{R}^{2} \bigr) \bigr) \\ &n(x,t)\in L^{\infty}\bigl(0,T;H^{l+2} \bigl( \mathbf{R}^{2} \bigr) \bigr),\qquad n_{t}(x,t)\in L^{\infty}\bigl(0,T;H^{l} \bigl(\mathbf{R}^{2} \bigr) \bigr) \\ &n_{tt}(x,t)\in L^{\infty}\bigl(0,T;H^{l-2} \bigl( \mathbf{R}^{2} \bigr) \bigr). \end{aligned}$$
